# Vibratome sectioning of tumors to evaluate the interactions between nanoparticles and the tumor microenvironment ex-vivo

**DOI:** 10.3389/fbioe.2022.1007151

**Published:** 2022-09-23

**Authors:** Shuhan Liu, Juechao Zhang, Ning-Ning Zhang, Xiandi Meng, Kun Liu, Yong-Guang Yang, Tianmeng Sun, Liguang Sun

**Affiliations:** ^1^ Key Laboratory of Organ Regeneration and Transplantation of Ministry of Education, The First Hospital, Jilin University, Changchun, China; ^2^ National-local Joint Engineering Laboratory of Animal Models for Human Diseases, Changchun, China; ^3^ State Key Laboratory of Supramolecular Structure and Materials, Jilin University, Changchun, China; ^4^ International Center of Future Science at Jilin University, Changchun, China

**Keywords:** vibratome sectioning, TME (tumor microenvironment), nanocarrier distribution, tumor-infiltrating macrophages, tumor-infiltrating dendritic cells

## Abstract

Nanoparticles have been investigated as drug carriers and promising agents for cancer therapy. However, the tumor microenvironment (TME), which is formed by the tumor, is considered a barrier for nanocarriers to enter the internal tumor tissue. Therefore, the evaluation of the biological distribution of nanocarriers in TME can provide useful information on their role in tumor-targeted drug delivery. Although the tumor-bearing mouse model is commonly used to investigate the distribution of nanocarriers in the TME, there is currently a lack of a testing system to predict the distribution of nanocarriers in tumor tissues, especially in patients. This study revealed that the macrophages and dendritic cells (DCs) were more distributed in the peripheral part than the central part of the tumor, which might be an obstacle to the uniform distribution of nanoparticles in the tumor. In addition, the cellular uptake of gold nanoparticles (AuNR and AuNS) in macrophages and DCs cell lines (RAW264.7 and DC1.2) was markedly different from that in the TME. Hence, the study model of the interaction between nanoparticles and macrophages and DCs has an important impact on the accuracy of the results. The vibratome sections of tumor tissues preserved the spatial distribution of immune cells and tumor cells, and had very little effects on their morphologies and activities. More importantly, we found that the distribution of nanocarriers in vibratome sections was similar to that in tumors *in vivo*. In all, *ex vivo* analysis using vibratome sections of tumor tissues provides a more convenient and stable method for elucidating the influences of TME on the distribution of nanocarriers.

## 1 Introduction

Nanoparticles as drug carriers have been designed through various modifications, such as altering their size, shape, and chemical and physical properties, for the efficient transport of drug molecules against cancer (Sun et al., 2014). However, the tumor microenvironment (TME), including blood vessels, immune cells, fibroblasts, and the extracellular matrix, usually forms a barrier for nanocarriers (Hanahan and Weinberg, 2011; Hanahan and Coussens, 2012; Joyce and Fearon, 2015; Wang et al., 2017; Xiong et al., 2019). TME plays an important role in the occurrence, development, invasion and metastasis of tumors (Barar and Omidi, 2013; Rostamizadeh et al., 2022). In addition, the irregular micro blood vessels and abnormal stroma of solid tumors have important pathophysiological barrier functions for cancer treatment, so the new strategy should target the biological elements of the TME (Omidi and Barar, 2014). In our previous study, we found that tumor-infiltrating immune cells, especially macrophages and dendritic cells (DCs), had formed a barrier for nanoparticles to reduce their efficiency for the delivery of tumor cell-targeted drugs (Wu et al., 2017). Therefore, it is necessary to develop a method through which we can assess the effects of tumor-infiltrating immune cells on the distribution of nanocarriers in tumors.

In contrast to the *in vitro* cell culture model, where the cells grow in a dish with optimized growth medium, the biological environment in the tumor is markedly more complicated (Qi et al., 2016; Musetti and Huang, 2018). Therefore, evaluating the biodistribution of nanocarriers in the TME provides useful information on the efficiency of nanocarriers in tumor-targeted drug delivery (Overchuk and Zheng, 2018; Stylianopoulos et al., 2018a; Stylianopoulos et al., 2018b). Currently, the cellular distribution of nanocarriers in the TME is commonly explored by flow cytometry and immunohistochemical staining of tumor sections from tumor-bearing mice receiving nanocarriers injections (Miao et al., 2017; Hombach-Klonisch et al., 2018; Hou et al., 2018; Qian et al., 2019). However, it is impossible to perform such measurements in patients. Hence, it is necessary to develop a method which can evaluate the distribution of nanocarriers in tumors *ex vivo*.

Both the activity and spatial distribution of cells within tumor tissues play important roles in the interaction with nanocarriers accumulated in the tumor. Therefore, we cannot evaluate the effects of TME on the ability of nanocarriers for tumor cell-targeted drug delivery through flow cytometry or the traditional slicing techniques using frozen or paraffin-embedded sections. Vibratome sectioning, which does not involve the use of any harsh organic solvents, can preserve the cell activity and the TME in the sections for a certain period of time (Clérin et al., 2014; Abdelaal et al., 2015; Iulianella, 2017). Vibratome tumor sections have been used to assess the effects of drugs on living tumor cells *ex vivo* (Gerlach et al., 2014; Koh et al., 2016; Roelants et al., 2020). Consequently, this multi-functional approach may provide an intuitive platform for the characterization of the distribution of nanocarriers within tumors. However, thus far, there are no research studies demonstrating that the interactions of nanocarriers with the TME can be performed on the vibratome tumor sections *ex vivo*.

Here, the cellular uptake of two types of gold nanoparticles [gold nanospheres (AuNS) and gold nanorods (AuNR)] by tumor-infiltrating macrophages (TIMs) and DCs (TIDCs) was observed. We found that the cells cultured in dishes cannot be used as a substitute for tumors *in vivo*. While, the distribution of AuNS and AuNR in vibratome tumor sections was evaluated *ex vivo*, and obvious accumulation of Cy5-AuNR or Cy5-AuNS in the tumor-infiltrating immune cells was observed, which was similar to the distribution noted in the *in vivo* experiment.

## 2 Materials and methods

### 2.1 Chemicals, antibodies, and cells

Dulbecco’s modified eagle medium (DMEM), penicillin/streptomycin, L-glutamine, fetal bovine serum (FBS), collagenase Type IV and 0.25% trypsin were all purchased from Thermo Fisher Scientific (Waltham, MA, United States). DNase I was purchased from Roche (New York, NY, United States). Hexadecyltrimethylammonium bromide (CTAB, ≥ 99%), gold (III) chloride trihydrate (HAuCl_4_·3H_2_O, 99.99%), sodium citrate tribasic dehydrate (citrate, ≥ 99%), sodium borohydride (NaBH_4_, ≥ 98%), L-ascorbic acid (AA, ≥ 99.0%), silver nitrate (AgNO_3_, ≥ 99.0%), and (3-aminopropyl) trimethoxysilane (APTMS, 97%) were purchased from Sigma-Aldrich. Fluorescence-labeled anti-mouse CD3 (145-2C11), CD4 (GK1.5), CD8 (53-6.7), CD11b (M1/70), CD11c (N418), CD19 (6D5), CD45 (30-F11), F4/80 (RM8), Ly-6C (104), Ly-6G (RA3-6B2), and NK1.1 (PK136) monoclonal antibodies were obtained from Biolegend (San Diego, CA, United States) and BD Biosciences (Franklin Lakes, NJ, United States). These antibodies were either unlabeled or conjugated with FITC, PE, PerCP-Cy5.5, PE-Cy7, APC, AF700, APC-Cy7, Pacific Blue, or BV605 as indicated.

The mouse melanoma cell line B16 cells, mouse dendritic cell (DC) line DC1.2 cells, and mouse macrophage cell line RAW264.7 cells were purchased from American Type Culture Collection (ATCC). The cells were cultured in DMEM supplemented with 10% FBS at 37°C incubator with 5% CO_2_. The cells were passaged 2-3 times per week with light trypsinization.

### 2.2 Synthesis of gold nanospheres and nanorods

The CTAB capped gold nanospheres (AuNSs) with average diameter of 31.55 nm ± 0.44 nm were synthesized based on the seed-mediated growth method reported by Murphy and co-workers (Ziegler and Eychmüller, 2011). Briefly, A 20 ml solution containing 0.25 mM HAuCl4 and 0.25 mM sodium citrate was prepared, and ice-cold NaBH4 (0.60 ml, 0.10 M) solution was added to the solution to get seeds. The seeds can be used after storing at 25°C for 2 h–5 h. Growth solution was the mixture of CTAB (0.3645 g in 8.2 ml H2O), HAuCl4 (0.60 ml, 15 mM), ascorbic acid (0.20 ml, 0.10 M). The 1.0 ml of seed was added into growth solution and stirred for 10 min to get 11 nm AuNSs. Next, the aqueous solutions of CTAB (8.5 ml, 0.10 M), HAuCl4 (0.167 ml, 15 mM), ascorbic acid (0.050 ml, 0.10 M) were mixed, and then added 1.5 ml of 11 nm AuNSs and stirred for 10 min. Briefly, AuNRs were prepared by mixing CTAB (3.5 ml, 0.10 M), HAuCl4 (0.12 ml, 15 mM), and ice-cold NaBH4 solution (0.50 ml, 10 mM) at 25.5°C. The solution was stirred for 2 min and stored at 25.5°C for 0.5 h–2 h to get seeds. The solutions of HAuCl4 (0.50 ml, 15 mM), AgNO3 (0.40 ml, 4.0 mM), and ascorbic acid (0.124 ml 0.079 M) were added into CTAB solution (0.3645 g in 8.86 ml H2O), and then added 0.10 ml seed solution. The mixture was incubated at 27.0°C for 12 h to obtain AuNRs. The gold nanorods (AuNRs) with an average length of 32.14 nm ± 0.77 nm and diameter of 8.57 nm ± 1.27 nm were prepared using the procedure reported by Keul and co-workers (Keul et al., 2007). We conjugate fluorescent dye on the surface of AuNSs and AuNRs after PEGylated. After addition of 1 ml Amine-PEG-thiol (MW 5,000, 0.12 mM) mixture to 5 ml of AuNSs or AuNRs (the concentration of AuNSs or AuNRs was concentrated to 15.0 nM), the mixture was stirred for 2 h at 24°C to allow complete conjugation *via* an Au-S bond. The mixture was then purified by centrifugation (AuNR, 11,500 rpm; AuNS, 7,500 rpm for 15 min at 20°C). The supernatant was removed, and the pellet was resuspended in PB solution (pH 8.4). Then, the pellet was resuspended in 1 ml of Cy5-NHS or AF488-NHS (10 μM) in PB solution (pH 7.4). After resuspension, the mixture was shaken for 2 h, and then purified by centrifugation. The supernatant was removed and the pellet was resuspended in deionized water and kept at 4°C in the dark.

### 2.3 Animals and tumor models

C57BL/6 mice were purchased from Beijing Vital River Laboratory Animal Technology Co., (Beijng, China). GFP transgenic C57BL/6-GFP mice (6 weeks–8 weeks) were purchased from Nanjing Biomedical Research Institute of Nanjing University (Nanjing, China). The animals were raised in a specific pathogen free environment with free access to food and water. All animals received care in compliance with the guidelines outlined in the Guide for the Care and Use of Laboratory Animals. All procedures were approved by the Jilin University Animal Care and Use Committee. To set up the B16 tumor-bearing mouse model, C57BL/6 mice were subcutaneously injected with 2 × 10^6^ B16 cells into the right side of the back.

The B16 tumor-bearing mice were administrated with AuNRs or AuNSs (10 pmol per mouse) by i.v., injection on day 14 after tumor cells implantation. Tumors were harvested at 24 h after AuNRs or AuNSs injection and measured the fluorescent signal of Cy5 or AF488 using Xenogen IVIS Lumina system (Caliper Life Science, Alameda, CA). Results were analyzed using Living Image^®^ 3.1 software (Caliper Life Sciences, Alameda, CA).

### 2.4 Flow cytometer analysis

Tumors were harvested from tumor-bearing mice at 24 h after i.v., injection of PBS, AuNRs or AuNSs, cut into small pieces, and digested into single cells using collagenase IV solution (200 mg/ml) containing DNase I (0.2 mg/ml). These cells were centrifuged at 1,650 rpm for 5 min, re-suspended in FACS buffer (PBS containing 0.1% BSA). All samples were filtered through a 40-μm nylon mesh filter and stained with fluorochrome-conjugated mAb against respective surface antigens: anti-CD3, anti-CD4, anti-CD8, anti-CD11b, anti-CD11c, anti-CD19, anti-CD45, anti-F4/80, anti-Ly-6C, anti-Ly-6G, anti-F4/80 and anti-NK1.1. All samples were collected on a fluorescence-activated cell sorter (LSR Fortessa, Becton Dickinson) and analyzed using Flowjo software (TreeStar). The single cell suspension of digested tumor tissue was harvested and stained for the following immune cell populations and markers: CD3^+^CD4^+^CD8^−^ (helper T cells), CD3^+^CD4^−^CD8^+^ (cytotoxic T cells), CD19^+^ (B cells), NK1.1^+^ (NK cells), CD11c^+^ (DCs), CD11b^+^F4/80^+^ (macrophages), Ly-6G^+^ (granulocytes) and CD11b^+^Ly-6G^−^Ly-6C^hi^ (monocytic myeloid derived suppressor cells, M-MDSCs).

### 2.5 Cellular uptake study

DC1.2 or RAW264.7 cells were seeded in 24-well plates at a density of 5 × 10^4^ cells/well with 500 μl complete DMEM medium and incubated over night at 37°C incubator with 5% CO_2_. Cy5-AuNRs or AF488-AuNSs were added into each well. After incubation at 37°C for 0.5 h or 2 h, the cellular uptake of Cy5-AuNRs or AF488-AuNSs were analyzed by flow cytometry.

### 2.6 Fluorescence immunohistochemical staining

Tumors were harvested from tumor-bearing mice at 24 h after i.v., injection of PBS, AuNRs, or AuNSs, and fixed with 4% paraformaldehyde (PFA) buffer for 16 h. After dehydrating using a 30% saccharose solution for 48 h, these tumors were sectioned into 10-μm thick sections. For macrophage staining, these slides were washed with PBS for 3 times and stained with AF647 labeled anti-F4/80 antibodies at 4°C overnight. For DCs staining, these slides were stained with anti-CD11c antibodies at 4°C overnight, and followed by labeled secondary antibodies at room temperature for 1 h. All slides were stained with DAPI (4,6-diamino-2-phenyl indole, Sigma). These slides were observed and photographed with a CLSM using a 20 × objective (LSM 880, Carl Zeiss, Oberkochen, Germany).

### 2.7 Vibratome section

Tumor tissues harvested from tumor-bearing mice at 24 h after i.v., injection of PBS, AuNRs, or AuNSs were embedded in low melting-temperature molten agarose II (Amresco, Atlanta, GA, United States), and sectioned using a V1000 vibratome (Leica, Germany) with 0.2 mm/s sectioning speed, 1.4 mm amplitude and 400-μm thickness. The tissue sections were cultured in complete DMEM medium containing Cy5-AuNRs (0.3 nM) or Cy5-AuNSs (0.3 nM) at 37°C for 2 h.

### 2.8 Statistical analysis

Data are represented as mean ± standard error of mean. Differences were analyzed using one-way analysis of variance (ANOVA) with post hoc Tukey’s test. A *p* value of < 0.05 was considered statistically significant.

## 3 Results

### 3.1 TIMs and DCs are mostly distributed in the tumor periphery

**FIGURE 1:**
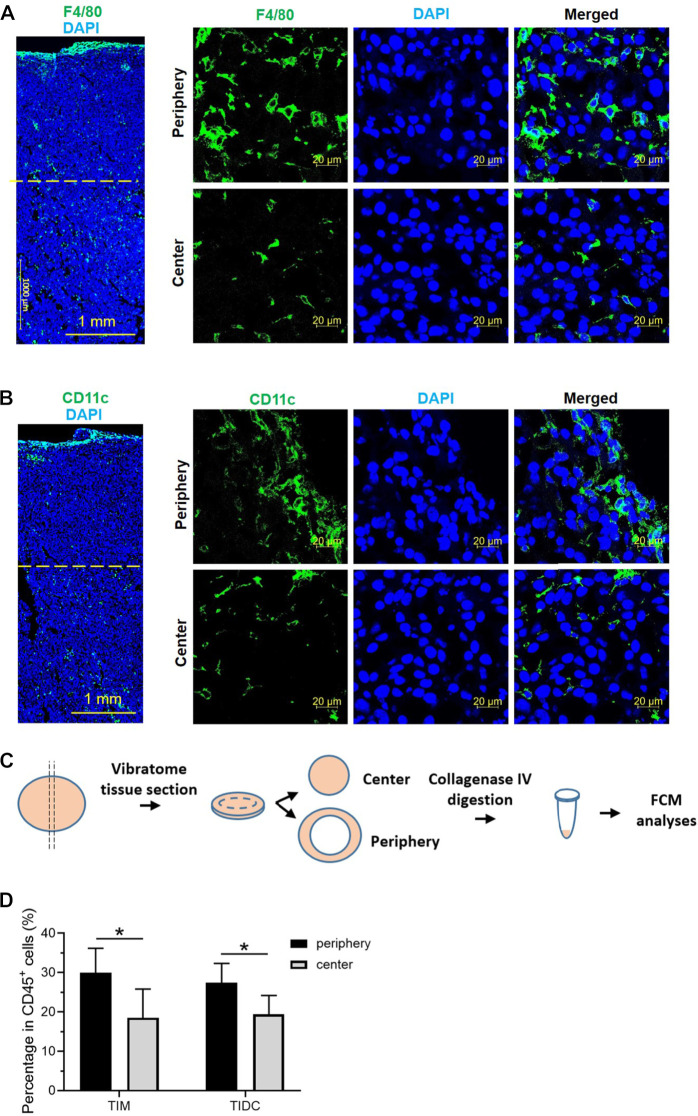
The tumor-infiltrating macrophages and DCs mostly distributed in the tumor periphery. **(A,B)** Confocal laser scanning microscopy (CLSM) images showed the distribution of macrophages **(A)** or dendritic cells **(B)** in B16 tumor tissue 14 days after subcutaneous tumor inoculation. The macrophages were stained with PE labeled anti-F4/80 antibody (green). The DCs were stained with APC labeled anti-CD11c antibody (green). The nuclei were stained with DAPI (blue). Scale bar, 1,000 μm (Large area image) or 20 μm (peripheral region and central region). **(C)** Schematic illustrations of vibratome tissue section and separation of B16 tumors into peripheral and central part. The tumor tissue was digested and flow cytometry was used to assess the tumor-infiltrating immune cells. **(D)** The frequency of tumor-infiltrating macrophages and DCs in CD45^+^ cells in the peripheral and central region of B16 tumor 14 days after subcutaneous tumor inoculation. Data are presented as mean ± SD (*n* = 5 per group). *p < 0.05.

Studies have shown significant differences in the TME between the tumor periphery and tumor center (Aishima et al., 2008; Pina et al., 2009). To confirm the unequal distribution of tumor-infiltrating immune cells within the tumor tissue, we observed the distribution of macrophages and DCs (two major mononuclear phagocytes) by immunofluorescence staining in a melanoma tumor-bearing mouse model. According to previous studies (Stylianopoulos et al., 2018a), we separated the tumor tissues into the tumor periphery, a 2 mm-wide band of tumor closely adjacent to the invasive front, and tumor center. As shown in Figures 1A,B; Supplementary Material S1, S2, both TIMs (F4/80^+^) and TIDCs (CD11c^+^) were widely distributed in the tumor periphery and tumor center, especially near the tumor edge. We further evaluated the abundance of TIMs and TIDCs among tumor-infiltrating immune cells (CD45^+^) in the center and periphery of the B16 tumor tissue. The 200 μm-thick tumor sections with the largest diameter were separated into peripheral and central tumor tissues, and the tumor-infiltrating immune cells were assessed by flow cytometry (Figure 1C; Supplementary Figure S3). The frequency of TIMs among CD45^+^ cells in the tumor periphery was significantly higher than that recorded in the tumor center, accounting for nearly 30% of CD45^+^ cells in the periphery and 18% in the central part (Figure 1D). Meanwhile, similar findings were found in TIDCs, accounting for 27% of CD45^+^ cells in the peripheral part and nearly 20% in the central part of tumor (Figure 1D). Other tumor-infiltrating immune cells, such as CD4^+^ T cells, CD8^+^ T cells, B cells, NK cells, M-MDSCs and granulocytes (Ly-6G^+^) were also evaluated in the peripheral and central part of B16 tumors (Supplementary Figure S4). And CD8^+^ T cells and NK cells were more distributed in the peripheral part of the tumor than the central part (Supplemetary Figure S4). These data demonstrated that the distributions of TIMs and TIDCs in B16 tumors differ between tumor periphery and tumor center, and there were more tumor-infiltrating myeloid cells in the peripheral part.

### 3.2 TIMs and TIDCs affect the distribution of nanoparticles within tumors

**FIGURE 2:**
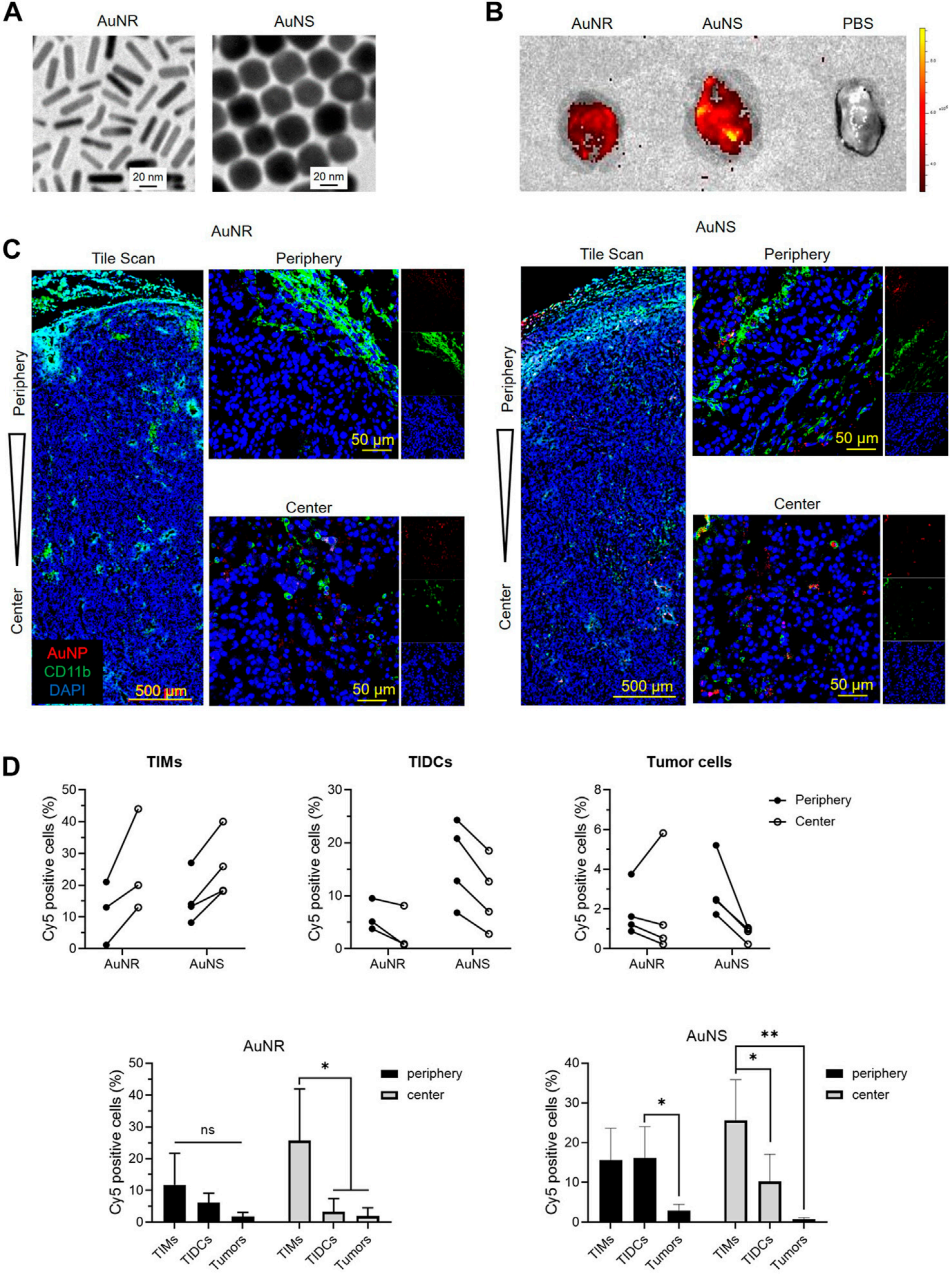
The TIMs and TIDCs affect the distribution of nanoparticles within tumors. **(A)** Representative of transmission electronic microscopic images of AuNR and AuNS. The scale bar is 20 nm. **(B)** Fluorescent images of B16 tumors harvested at 24 h after intravenously injection of PBS, Cy5-AuNR or Cy5-AuNS. **(C)** CLSM images show the tile scan images of periphery and center of B16 tumors challenged with Cy5-AuNR or Cy5-AuNS (red). Tumor-infiltrating myeloid cells were stained with CD11b-PE (green). Cell nuclei were stained with DAPI (blue). **(D)** Cellular uptake of Cy5-AuNR and Cy5-AuNS by TIMs, TIDCs or tumor cells in the periphery or center region in B16 tumors measured by flow cytometry (top panel). Statistical analysis of Cy5 positive cells of TIMs, TIDCs or tumor cells (bottom panel). The scale bar is 500 μm (tile scan) or 50 μm (amplified images of periphery and center).

To evaluate the influences of TIMs and TIDCs on the distribution of nanoparticles in tumor tissues, we prepared PEGylated AuNR and AuNS with similar size as previously described (Black et al., 2014), which are the most commonly investigated metallic nanoparticles. The transmission electron microscopy images (Figure 2A) indicated that the AuNS had a spherical shape with an average size of ∼30 nm; the mean length and width of AuNR were approximately 30 nm and 8 nm, respectively. The Zeta potential of Amine-PEG-thiol modified AuNSs and AuNRs was +8.39 mV and +5.95 mV, respectively (Supplemetary Figure S5A). Due to the amino groups were weakly positively charged, the AuNS@PEG-NH_2_ and AuNS@PEG-NH_2_ were also weakly positively charged. And the size of Amine-PEG-thiol modified AuNSs and AuNRs was 77.74 nm and 87.78 nm, respectively (Supplementary Figure S5B). We evaluated the biodistribution and accumulation of fluorescent dye (Cy5)-labeled AuNR and AuNS in B16 tumor tissue. Cy5-AuNR or Cy5-AuNS were administered by i.v., injection 14 days after B16 cell inoculation. The distribution of Cy5-AuNR or Cy5-AuNS in tumor tissues was measured 24 h after i.v., injection by confocal laser scanning microscopy (CLSM) and flow cytometry. As shown in Figure 2B, both Cy5-AuNR and Cy5-AuNS showed obvious accumulation in tumor tissues. Furthermore, the fluorescent signal of Cy5 in Cy5-AuNS-treated tumors was higher than that observed in Cy5-AuNR-treated tumors, which was consistent with previous reports (Black et al., 2014). In addition, significant accumulation of Cy5-AuNS and Cy5-AuNR was found in the kidney, liver, and spleen (Supplementary Figure S6). The cellular distribution of Cy5-AuNR and Cy5-AuNS in tumor tissues was further assessed by CLSM and flow cytometry. We stained the tumor-infiltrating myeloid cells, including TIMs and TIDCs, using an anti-CD11b antibody. The CLSM images indicated that both Cy5-AuNR and Cy5-AuNS were widely distributed in the peripheral and central area of B16 tumors (Figure 2C). The CLSM images showed that Cy5-AuNR obviously colocalized with CD11b^+^ cells in both the tumor periphery and tumor center (Figure 2C). A significant distribution of fluorescent signal in the cytoplasm of tumor-infiltrating myeloid cells was also observed in Cy5-AuNS-treated tumors (Figure 2C). Quantitative evaluation of the cellular uptake of Cy5-AuNR and Cy5-AuNS by TIMs, TIDCs, and tumor cells was further performed by flow cytometry. As shown in Figure 2D, the frequency of Cy5^+^ TIDCs in the tumor periphery was higher than that noted in the tumor center after i.v., injection of Cy5-AuNR or Cy5-AuNS. Nevertheless, the TIMs in the tumor center showed an enhanced ability to engulf Cy5-AuNR or Cy5-AuNS. There was similar distribution of Cy5-AuNR between the CD45^−^ tumor cells in the tumor periphery and tumor center. However, a markedly higher percentage of Cy5^+^CD45^−^ tumor cells was found in the periphery of Cy5-AuNS-treated tumors. In addition, both TIMs and TIDCs exhibited stronger abilities to engulf AuNR and AuNS than tumor cells. These data suggest that the tumor-infiltrating immune cells become a barrier for the tumor cell-targeted drug-loaded nanoparticles.

### 3.3 Macrophages and DCs cultured *in vitro* are not suitable for studying the interactions between nanoparticles and TIMs and TIDCs

**FIGURE 3:**
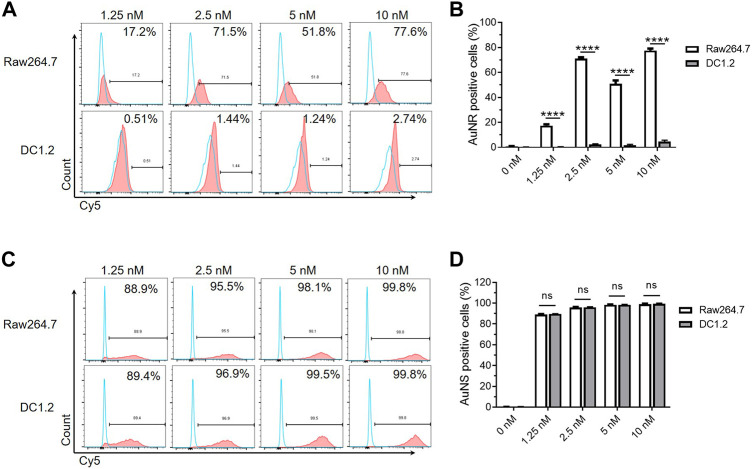
The macrophages and DCs cultured *in vitro* are not suitable for studying the interactions between nanoparticles and TIMs and TIDCs. **(A)** Flow cytometry analyses of AuNR accumulation in DC1.2 and Raw264.7 cell lines (*n* = 3). The concentration of Au nanoparticles was 1.25 nM, 2.5 nM, 5 nM and 10 nM. **(B)** Frequencies of AuNR positive cells in DC1.2 and Raw264.7 cell lines 2 h after AuNR challenge at 37°C (*n* = 3). **(C)** Flow cytometry analyses of AuNS accumulation in DC1.2 and Raw264.7 cell lines (*n* = 3). The concentration of Au nanoparticles was 1.25 nM, 2.5 nM, 5 nM and 10 nM. **(D)** Frequencies of AuNS positive cells in DC1.2 and Raw264.7 cell lines 2 h after AuNS challenge at 37°C (*n* = 3). ****p < 0.0001.

Traditional two-dimensional cell culture is an indispensable tool for basic research and a wide range of clinical *in vitro* studies. The DC1.2 and RAW264.7 cell lines have been used to study the interaction of murine DCs, macrophages, and nanoparticles *in vitro* (Hu et al., 2011; Binder et al., 2012). We further examined whether we can study the effects of TIMs and TIDCs on nanoparticles using these cell lines *in vitro*. The fluorescent signal of Cy5 in DC1.2 or RAW264.7 cells was measured by flow cytometry after being cultured with Cy5-AuNR or Cy5-AuNS at different concentrations for 2 h, respectively. As shown in Figure 3A, B, the frequency of Cy5^+^ cells in Cy5-AuNR-treated RAW264.7 cells was significantly higher than that reported in DC1.2 cells at the same concentration of Cy5-AuNR. This finding indicated that RAW264.7 cells have a stronger ability to internalize AuNR than DC1.2 cells. However, incubation with Cy5-AuNS resulted in a similarly high level of Cy5^+^ RAW264.7 cells and Cy5^+^ DC1.2 cells, accounting for more than 85% of total cells at the concentration of 1.25 nM or more (Figure 3C,D). The results suggested that cell lines cultured separately *in vitro* could not simulate the phagocytosis of myeloid cells in TME *in vivo*.

**FIGURE 4:**
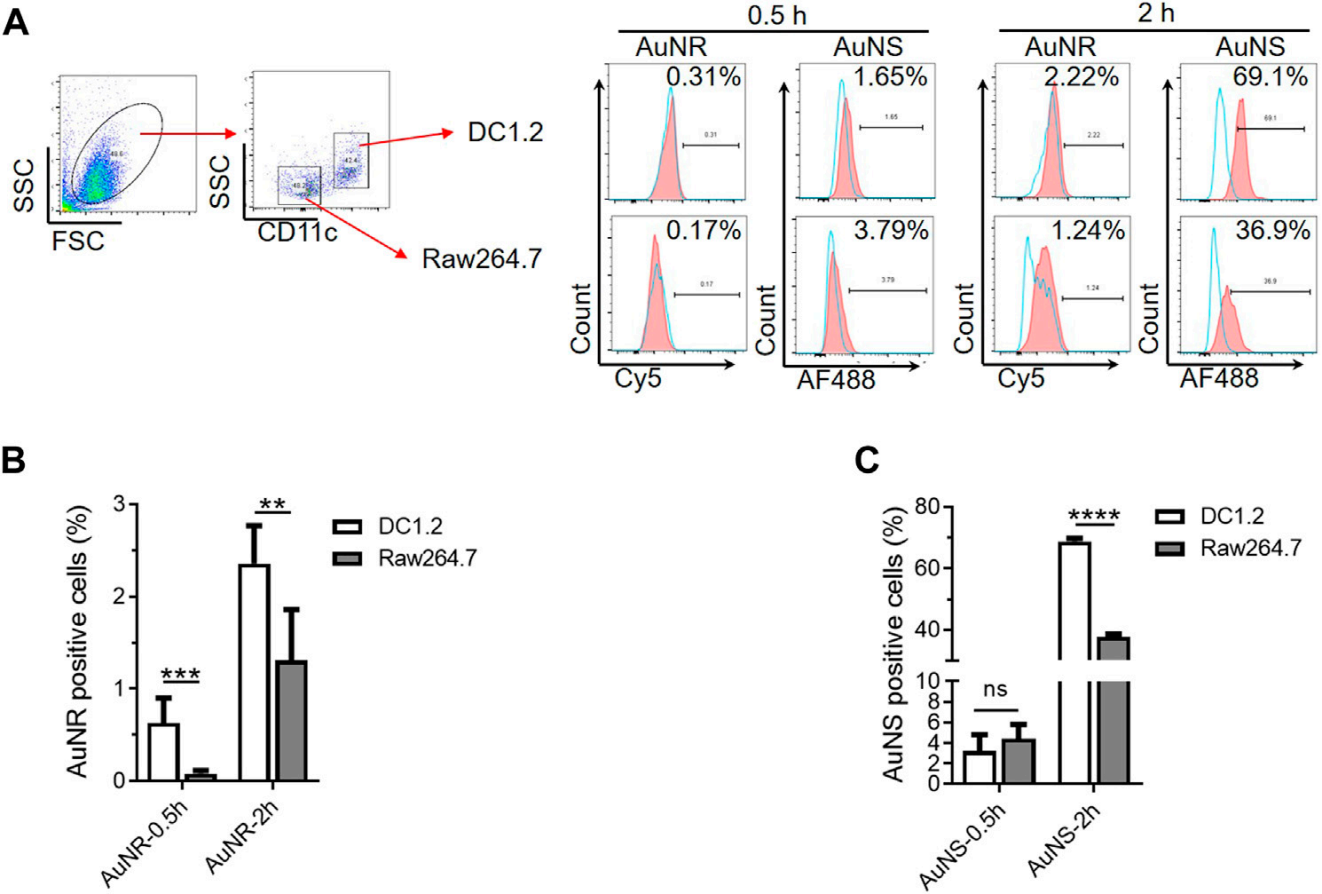
The co-culture system of macrophages and DCs *in vitro* are not suitable for studying the interactions between nanoparticles and TIMs and TIDCs. **(A)** Flow cytometry analyses of AuNR and AuNS accumulation 0.5 h and 2 h under DC1.2 and Raw 264.7 cell co-culture condition. **(B,C)** Statistical analysis of AuNR positive cells **(B)** or AuNS positive cells **(C)** under DC1.2 and Raw 264.7 cell co-culture system (*n* = 3). **p < 0.005, ***p < 0.0005, ****p < 0.0001.

The TIMs and TIDCs co-existed in the microenvironment of the tumor periphery and tumor center. Therefore, we further evaluated the cellular uptake of AuNR and AuNS by co-cultured DC1.2 and RAW264.7 cells. A mixture of DC1.2 cells and RAW264.7 cells at a ratio of 1:1 was cultured with the fluorescent dye-labeled AuNR (Cy5-AuNR) or AuNS (AF488-AuNS) for 0.5 h and 2 h. The fluorescent signal-positive DC1.2 cells (CD11c^+^) and RAW264.7 cells (F4/80^+^) were measured by flow cytometry (Figure 4A). As shown in Figure 4B, the frequency of Cy5^+^ DC1.2 cells was markedly higher than that of Cy5^+^ RAW264.7 cells at both time points. A similar cellular uptake of AF488-AuNS was found in DC1.2 cells and RAW264.7 cells after 0.5 h of culture (Figure 4C). Nevertheless, 68.77% of DC1.2 cells were AF488-positive after 2 h of culture with AF488-AuNS; this percentage was significantly higher than that determined for RAW264.7 cells (Figure 4C). These data indicate that the cellular uptake of nanoparticles by macrophages and DCs was influenced by the culture conditions *in vitro*. In co-culture condition, DCs might inhibit the phagocytosis of macrophages. Hence, it is difficult to evaluate the interactions between nanoparticles and macrophages and DCs in tumor tissues using the two-dimensional cell culture model.

### 3.4 Vibratome sections of tumor can be used to evaluate the distributions of AuNR and AuNS in tumors *ex vivo*

**FIGURE 5:**
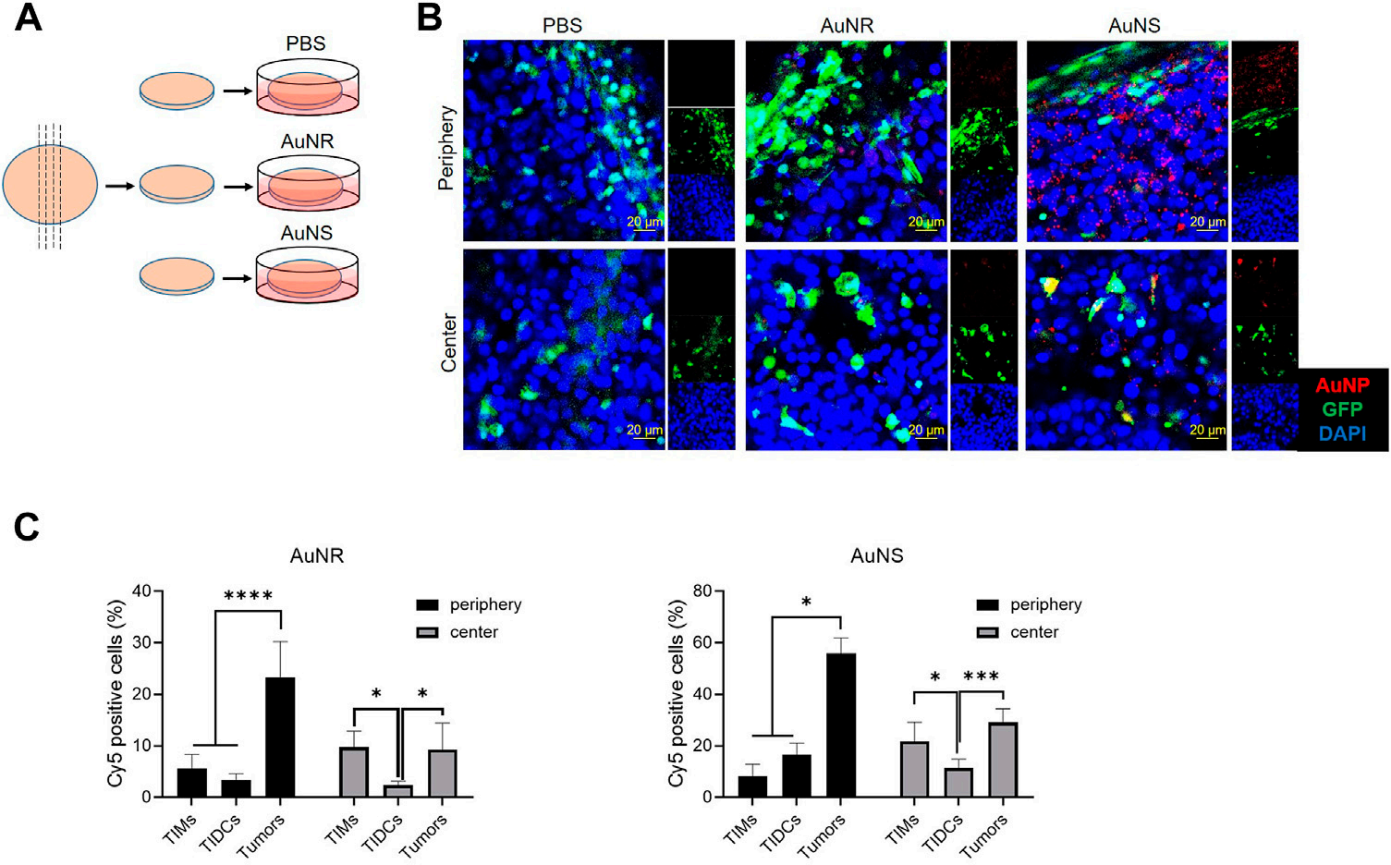
The vibratome sections of tumor can be used to evaluate the distributions of AuNR and AuNS in the tumor *ex vivo*. **(A)** Schematic illustrations of vibratome tissue section and *ex vivo* AuNP upake. The B16 tumor cells were subcutaneously implanted into GFP transgenic mice 14 days before the tumor was harvested. Afterwards, 200 μm-thick tumor vibratome sections were obtained and then incubated with PBS, Cy5-AuNR, or Cy5-AuNS at 37°C for 2 h **(B)** CLSM images of fluorescence signal in the tumor vibratome sections which was indicated in **(A)**. **(C)** Fluorescence signal in the tumor vibratome sections by flow cytometry. Cellular uptake of Cy5-AuNR and Cy5-AuNS by TIMs, TIDCs or tumor cells in the periphery or center region in B16 tumors of GFP mice measured by flow cytometry. Cy5 labeled AuNPs (red) were accumulated in the vibratome tumor sections of B16 tumor bearing GFP (green) mice. Cell nuclei were stained with DAPI (blue). The scale bar is 20 μm.

Sectioning with a vibrating microtome generates thick sections that are particularly useful for revealing histological and three-dimensional details in tissues. To evaluate whether the vibratome sections of tumors can be used to study the distribution of nanoparticles in tumor tissues *ex vivo*, we observed the fluorescent signal in such sections of tumors after incubation with Cy5-AuNS or Cy5-AuNR. The B16 tumor cells were subcutaneously implanted into green fluorescent protein (GFP) transgenic mice, and 200 μm-thick tumor vibratome sections were obtained 14 days after inoculation. After incubation with phosphate-buffered saline, Cy5-AuNR, or Cy5-AuNS at 37°C for 2 h, the fluorescence signal in the tumor vibratome sections was measured by CLSM (Figure 5A). As shown in Figure 5B, compared with the phosphate-buffered saline control, both Cy5-AuNR and Cy5-AuNS showed obvious accumulation in GFP^+^ cells in both the tumor periphery and tumor center, which was in line with the distribution of Cy5-AuNR and Cy5-AuNS in tumor tissues after i.v., injection (Figure 2C). However, cellular uptake of Cy5-AuNR and Cy5-AuNS by GFP^−^ tumor cells was also clearly observed in the sections (Figure 5B; Supplementary Figure S7), which was different from the findings of the *in vivo* experiment (Figure 2C). We also measured the fluorescence signal in the tumor vibratome sections by flow cytometry and found that the proportions of Cy5^+^ TIMs or TIDCs was similar to *in vivo* models. Moreover, the phagocytic capacity of tumor cells in the vibratome sections was higher than that *in vivo* tumor models, which was consistent with the CLSM images (Figure 5C). In other words, when tumor cells are directly exposed to nanoparticles, tumor cells can phagocytize nanoparticles. However, in the *in vivo* tumor models, the existence of TME hinders the tumor cells from phagocytizing nanoparticles. These data suggest that the TME, especially TIMs and TIDCs, formed a barrier for tumor cell-target delivery of nanoparticles. So that TME is important for evaluating the distribution of nanoparticles in tumors, and the thick vibratome section method provides a feasible approach to studying the properties of nanoparticles in tumors *in vitro*.

## 4 Discussion

The immune and inflammatory cells are important players in the TME. TME, the hallmark of solid tumors, is associated with uncontrolled process of cell growth, division, metastasis and progression in solid tumors, and pH is a crucial factor in TME. It has been reported that the dysregulation of pH in solid tumors facilitates cancer cells to escape from apoptosis, endowing them with aggressive and metastatic phenotypes, and resistance to chemotherapy or radiotherapy (Asgharzadeh et al., 2017). Both *in vitro* and *in vivo* models to investigate the TME have been available for a long time. The aim of these models is to develop effective therapies for improving the survival of patients with poor prognosis. Nanoparticles have been investigated as drug carriers and promising agents for cancer therapy. Tumor-infiltrating immune cells in the TME have a significant impact on the fate of nanocarriers in the tumor tissue. Therefore, investigating the distribution characteristics of nanocarriers in the TME may improve the efficiency of drug delivery. Although the accumulation of nanoparticles inside tumors is mainly caused by enhanced permeability and the retention effect, the relationship between the distribution of immune cells and the accumulation of nanoparticles inside tumor tissue has not been thoroughly investigated. This study showed that the distribution of immune cells in the TME is not uniform, and the phagocytic ability of macrophages and DCs in the tumor center and tumor periphery is also different after intravenous administration of nanoparticles. Therefore, TIMs and TIDCs affect the distribution of nanocarriers within tumors.

A high degree of consistency between the experimental model and target tumor tissue is essential for the development of nanocarriers. Most preclinical studies on nanoparticles are conducted by using established cell lines and mouse models, and flow cytometry and immunofluorescent staining are usually used to analyze the distribution of nanoparticles in the tumor after i.v., injection. Therefore, a large number of tumor-bearing mice are needed, leading to high experimental cost. Moreover, these traditional experimental methods can only be used in animal experiments, rather than tumors of clinical patients. More efficient and accurate methods to detect the distribution of nanocarriers in the TME are warranted.

Vibration sectioning is an ideal method for short-term primary culture. A vibrating knife is used to cut the tissue, resulting in lower mechanical impact. This process does not involve the use of any harsh organic solvents that damage cells; hence, it is suitable for processing samples stained with fluorescent antibodies or dyes. Most importantly, this technique preserves the viability of cells, and the living tissue can be sectioned for subsequent culture, operation, and *in vivo* imaging (Iulianella, 2017). Moreover, it may be applied to most types of solid tumors. Using this technique, the non-fixed live tumor tissues can be sliced and cultured directly for a period of time. This method shows the best comparability with the original tumor for preserving the TME and morphology; thus, the experimental success rate is high and the generation time is short (Martin et al., 2019). The thickness of the slices for *ex vivo* culture is determined to include all cell types of the tumor, allowing examination of the multicellular biochemical processes, such as metabolism, drug transport, and biotransformation, in an almost natural environment (de Graaf et al., 2010). Other applications of the tumor tissue slices include the analysis of tumor response to drugs and exploration of signal transduction pathways (Parajuli and Doppler, 2009; Naipal et al., 2016). This model was used mostly in human carcinomas for visualizing live-cell calcium response behavior in human parathyroid adenoma tumor cell responsiveness to extracellular calcium challenge (Koh et al., 2016) or assaying tumor angiogenesis and microglia in the brain (Ghoochani et al., 2016). This culture system also harbors great potential as a drug sensitivity testing system for the personalized treatment of numerous types of carcinoma, such as human head and neck squamous cell carcinoma (Gerlach et al., 2014), pancreatic ductal adenocarcinoma (Misra et al., 2019), renal carcinoma (Roelants et al., 2020), glioma (Ghoochani et al., 2016), and the hepatic metastatic tissue of colorectal carcinoma (Martin et al., 2019). Similarly, this model was also utilized in the investigation of normal organ tissue. For example, Gerpe et al. took advantage of the precision-cutting of lung slices to evaluate viruses and viral vectors for gene and oncolytic therapy (Rosales Gerpe et al., 2018).

There is some research on the application of nanoparticles in vibrating sections. Kersting et al. studied the penetration of graphene quantum dots into precision-cut mammary tumor tissue slices, and found that this model was far closer to the reality model system than monoclonal cell lines (Kersting et al., 2019). Hofmann et al. used the co-culture system with an organotypic lung slice to investigate the toxic effect of amorphous silica nanoparticles, concluding that it as a useful tool for research at the organoid level (Hofmann et al., 2015). However, its application to study the effect of TME on the delivery efficiency of nanocarriers has not been investigated. This study described an image-based approach for predicting the distribution of nanocarriers in the TME while preserving the native tumor tissue context. Compared with the animal tumor model *in vivo*, this *ex vivo* model shows various similarities in histology and cell biology in the TME. The results of the present study support the concept that, unlike the *in vitro* cell culture, the *ex vivo* tumor tissue slice model can closely simulate drug administration *in vivo*. Therefore, the vibration slicing technology can be used to investigate the distribution characteristics of nanocarriers in cells in the TME, providing a convenient evaluation strategy for the distribution of nanocarriers.

## 5 Conclusion

In summary, we have investigated the potential of using vibratome tumor sections to evaluate the distribution of nanocarriers in the TME *in vivo*. The macrophages and DCs in the TME significantly affected the distribution of nanocarriers. However, the macrophage and DC cell lines cultured in dishes were not appropriate substitutes for measuring the interactions of nanocarriers with TIM and TIDCs in the TME. The cell morphology and cell activity are maintained in vibratome sections, indicating that these sections can be used to study the TME *ex vivo*. The distribution of nanocarriers in vibratome tumor sections was similar to that observed *in vivo*. *Ex vivo* analysis of tumor tissue slices provides a more convenient and stable method for elucidating the distribution of nanocarriers in the TME, and closely resembles the *in vivo* environment.

Vibratome tumor section technique is a promising tool for exploring the distribution of nanoparticles, because it can efficiently reflect the TME and simulate the internal environment, and TME is a significant factor for nanoparticle distribution. The survival time of tissues prepared using the vibrating section technique in in vitro culture can only be maintained for a limited period of time, which is a challenge for clinical translation. However, since the phagocytosis of nanoparticles by immune cells and tumor cells occurs in a short time, the distribution of nanoparticles in tumors can be observed intuitively and simply, providing a very promising research tool for nanodrug delivery in human tumor tissues.

## Data Availability

The raw data supporting the conclusions of this article will be made available by the authors, without undue reservation.
