# Supplementary material for: Vibratome sectioning of tumors to evaluate the interactions between nanoparticles and the tumor microenvironment ex-vivo
Source: Front Bioeng Biotechnol. 2022 Sep 23;10:1007151. doi: 10.3389/fbioe.2022.1007151 (PMC9537459; doi:10.3389/fbioe.2022.1007151)
Supplement: Supplementary file 1 [file DataSheet1.docx]

Supplementary Material

## Supplementary Figures


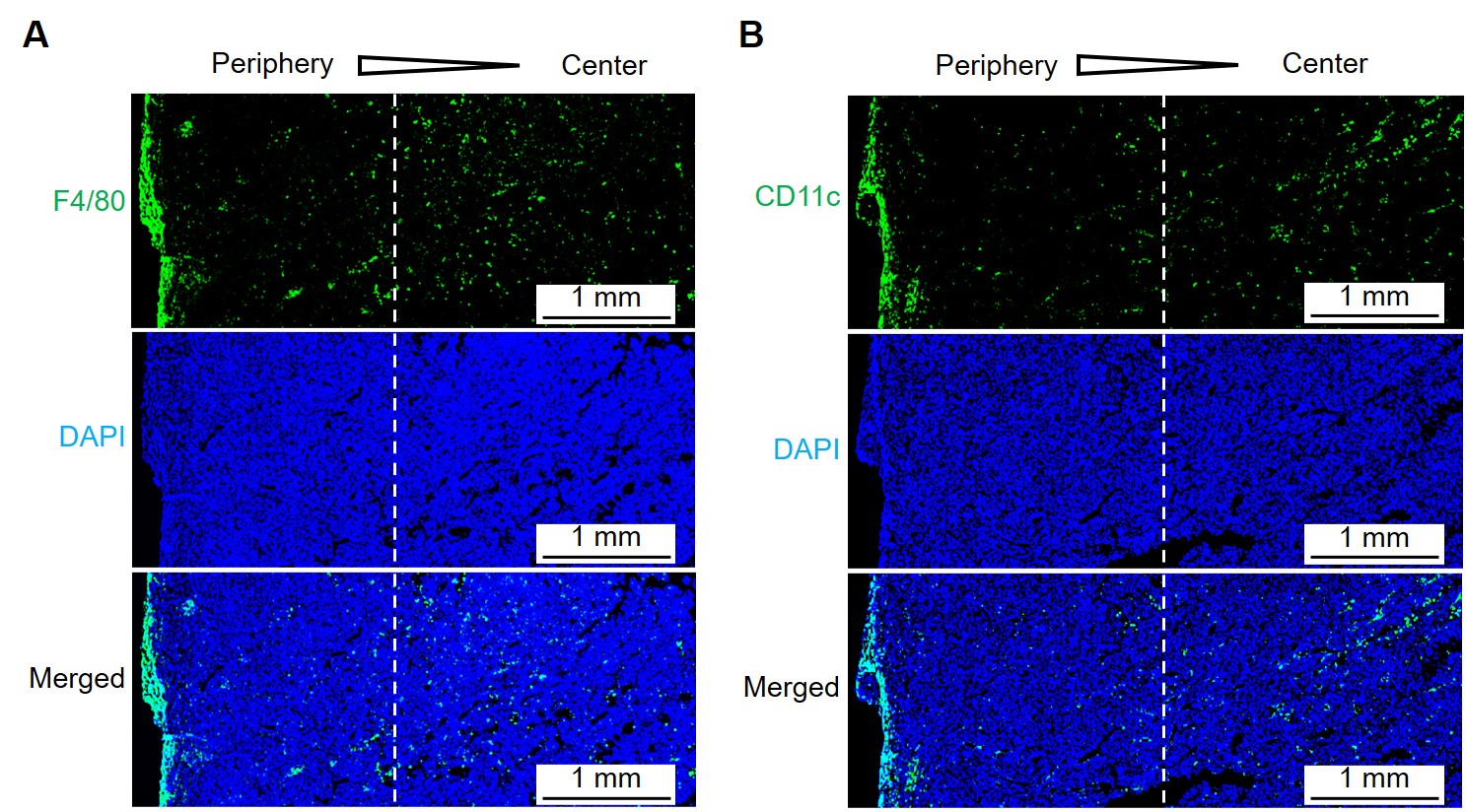


**Supplementary Figure S1.** The tumor-infiltrating macrophages and DCs mostly distributed in the tumor periphery. (A, B) CLSM images showed each color channel of the merged images in Figure 1A (A) and Figure 1B (B). The macrophages were stained with PE labeled anti-F4/80 antibody (green). The DCs were stained with APC labeled anti-CD11c antibody (green). The nuclei were stained with DAPI (blue). The scale bar is 1000 μm.


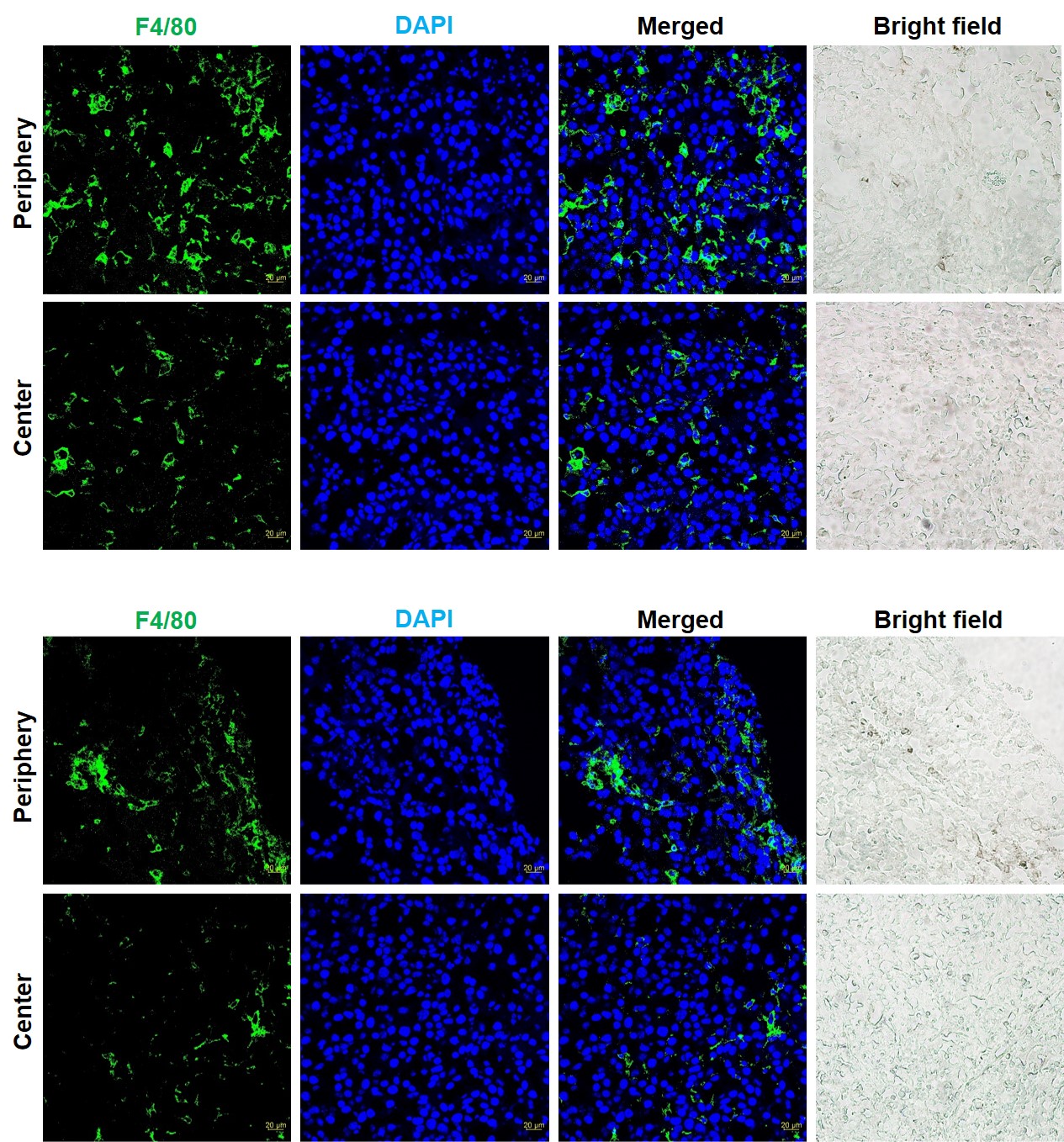


**Supplementary Figure S2.** Broadened vision and bright field of Figure 1A (top panel) and 1B (bottom panel). The scale bar is 20 μm.


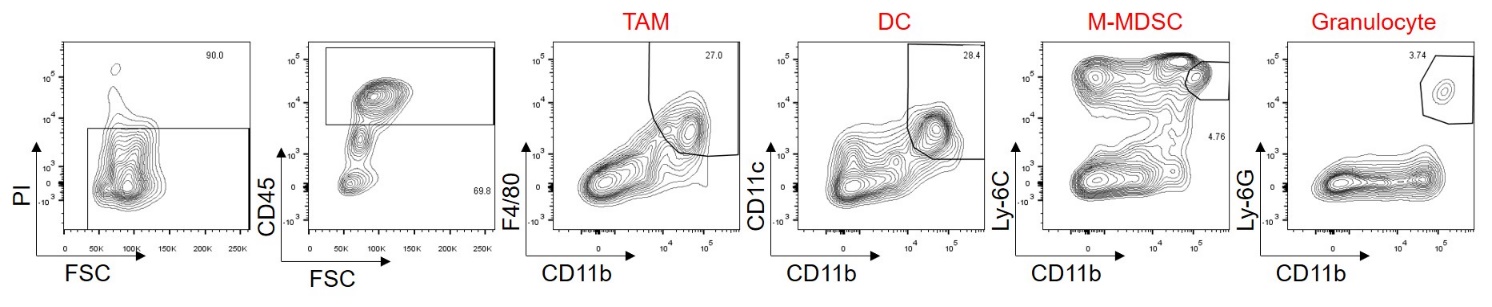


**Supplementary Figure S3.** Gating strategies of immune cells in the TME of B16 tumor tissue. Representative flow cytometry plots demonstrating the gating scheme for flow cytometry analysis of B16 tumor infiltrating-immune cells in TAM (CD11b^+^F4/80^+^), DC (CD11b^+^CD11c^+^), M-MDSC (CD11b^+^Ly-6C^+^) and granulocyte (CD11b^+^Ly-6G^+^).


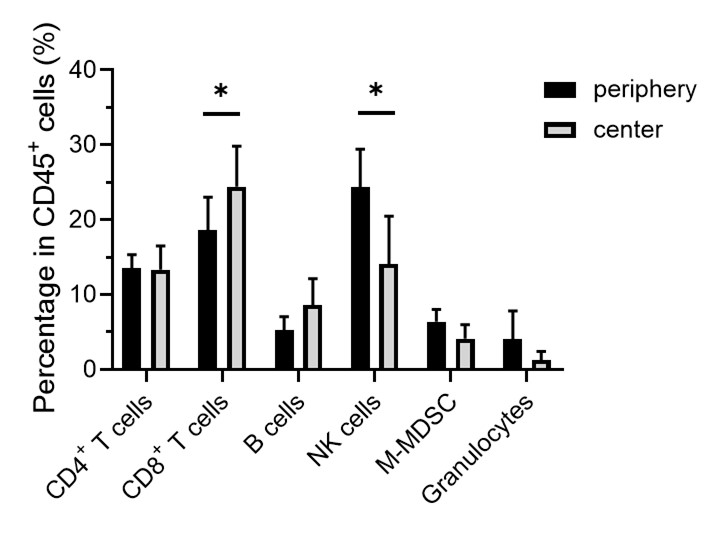


**Supplementary Figure S4.** The frequency of tumor-infiltrating CD4^+^ T cells, CD8^+^ T cells, B cells, NK cells, M-MDSCs and granulocytes in CD45^+^ cells in the peripheral and central region of B16 tumor 14 days after subcutaneous tumor inoculation. Data are presented as mean ± SD (n = 5 per group). *, p < 0.05.


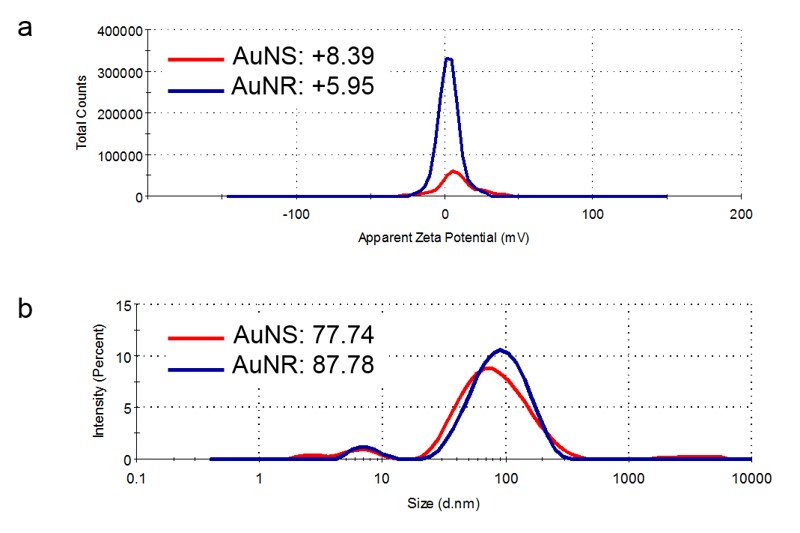


**Supplementary Figure S5.** (a) The Zeta potential of Amine-PEG-thiol modified AuNSs and AuNRs. (b) The size of Amine-PEG-thiol modified AuNSs and AuNRs.


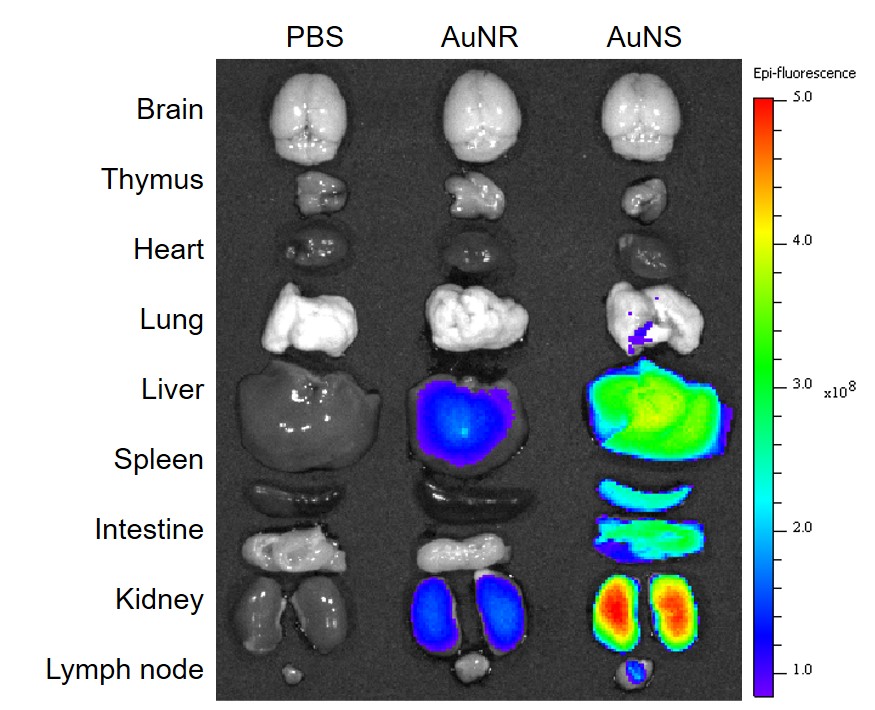


**Supplementary Figure S6.** Distribution of Cy5-AuNR or Cy5-AuNS within tumors. B16 tumor-bearing mice were administrated with PBS, Cy5-AuNR or Cy5-AuNS by *i.v.* injection. The Cy5 fluorescence images of indicated tissues harvested at 24 h after injection were conducted using IVIS Xenogen imaging system.


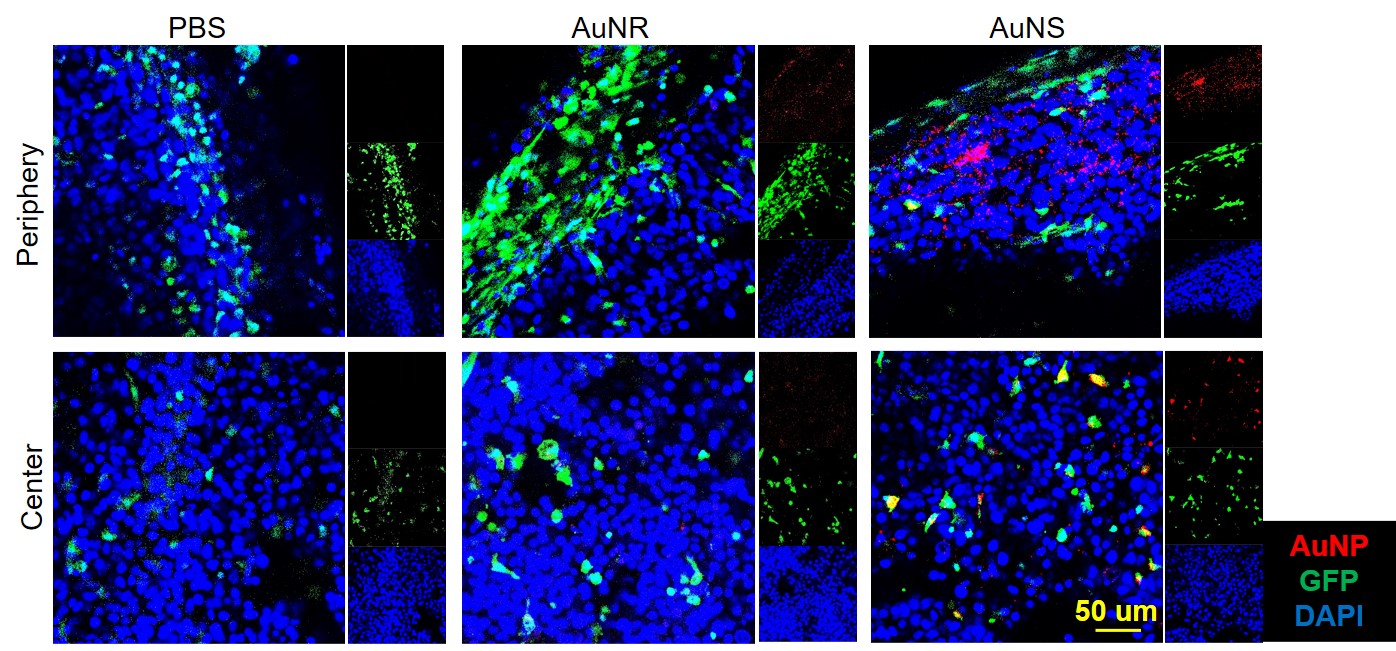


**Supplementary Figure S7.** Broadened vision of Figure 5B. The scale bar is 50 μm.
